# Identification of podocyte molecular markers in diabetic kidney disease via single-cell RNA sequencing and machine learning

**DOI:** 10.1371/journal.pone.0328352

**Published:** 2025-07-21

**Authors:** Hailin Li, Quhuan Li, Zuyan Fan, Yue Shen, Jiao Li, Fengxia Zhang

**Affiliations:** 1 School of Biology and Biological Engineering, South China University of Technology, Guangzhou, China; 2 First Affiliated Hospital of Gannan Medical University, Ganzhou, Jiangxi Province, China; University of Milan: Universita degli Studi di Milano, ITALY

## Abstract

Diabetic kidney disease (DKD) is a major cause of end-stage renal disease globally, with podocytes being implicated in its pathogenesis. However, the underlying mechanisms of podocyte involvement remain unclear. The aim of the present study was to identify podocyte molecular markers associated with DKD using single-cell RNA sequencing (scRNA-seq) data from patients with early DKD. Through enrichment analysis, subcluster clustering, and ligand–receptor (LR) interaction analysis, we elucidated the role of podocytes in early DKD progression. Podocyte heterogeneity and functional differences in DKD were observed. Multiple machine-learning algorithms were used to screen and construct diagnostic models to identify hub differentially expressed podocyte marker genes (DE-podos), revealing ARHGEF26 as a significantly downregulated marker in DKD. Validation using external datasets, reverse transcription quantitative real-time PCR (RT-qPCR) and Western blot confirmed it as a potential diagnostic biomarker. Our findings elucidate podocyte function in DKD and provide viable therapeutic targets, potentially improving diagnostic accuracy and treatment outcomes.

## Introduction

Diabetic kidney disease (DKD) is a diabetes-induced complication characterized by persistent albuminuria, glomerular basement membrane thickening, and progressive renal dysfunction [1]. It accounts for 30%–50% of end-stage kidney disease [2]. The global incidence of diabetes is increasing year by year [3]. The prevalence of DKD among people with diabetes is 20%–50% [4]. With the continuous increase in the incidence of DKD, it has emerged as a public health concern that threatens the health of a significant number of individuals worldwide. However, the pathogenesis of DKD is poorly understood.

Substantial research has been conducted recently to elucidate the pathogenesis of DKD. Podocytes are integral to the glomerulus and preserve the glomerular filtration barrier by interacting with the intracellular actin cytoskeleton through cell-adhesion molecules that adhere to the glomerular basement membrane (GBM), thereby preventing the loss of macromolecules [5]. During DKD development, the podocyte population undergoes progressive loss, manifested as the detachment of podocytes from the GBM [6]. In addition, proteinuria, a hallmark of renal diseases, including DKD, may stem from GBM alterations because of podocyte dysfunction [7,8]. Emerging evidence highlights the critical vulnerability of podocytes to circulating proteins, suggesting that podocyte apoptosis is implicated in the development of DKD [9]. Podocytes also contribute to the maintenance of glomerular endothelial cell function, which is crucial for the filtration barrier [10]. Therefore, the occurrence of GBM abnormalities is related to the cellular communication between podocytes and endothelial cells [11]. However, the specific mechanisms by which podocytes participate in DKD progression remain unclear.

In recent years, microarray analyses have shed light on the genetic underpinnings of DKD, revealing an increasing number of key genes that could serve as potential biomarkers for this condition [12,13]. Additionally, single-cell RNA sequencing (scRNA-seq) enables precise examination of individual cell transcriptomes, capturing their states at specific times or after interventions [14], thereby offering high-resolution insights into cellular heterogeneity and treatment responses. In the field of kidney disease investigation, scRNA-seq has been applied to better understand kidney homeostasis, development, and damage [15,16], such as in renal cell carcinoma [17], diabetic nephropathy [18], and IgA nephropathy [19]. Previously, we conducted research on DKD, utilizing bulk RNA sequencing (bulk RNA-seq) at the transcriptome level, and identified a biomarker in the glomeruli [20]. However, the limitations inherent to bulk RNA-seq, particularly its inability to resolve gene expression at the level of individual cell types, have constrained our understanding of cellular heterogeneity in glomeruli. Therefore, integration of scRNA-seq with bulk RNA-seq is essential for comprehensive transcriptome analysis.

**Fig 1:**
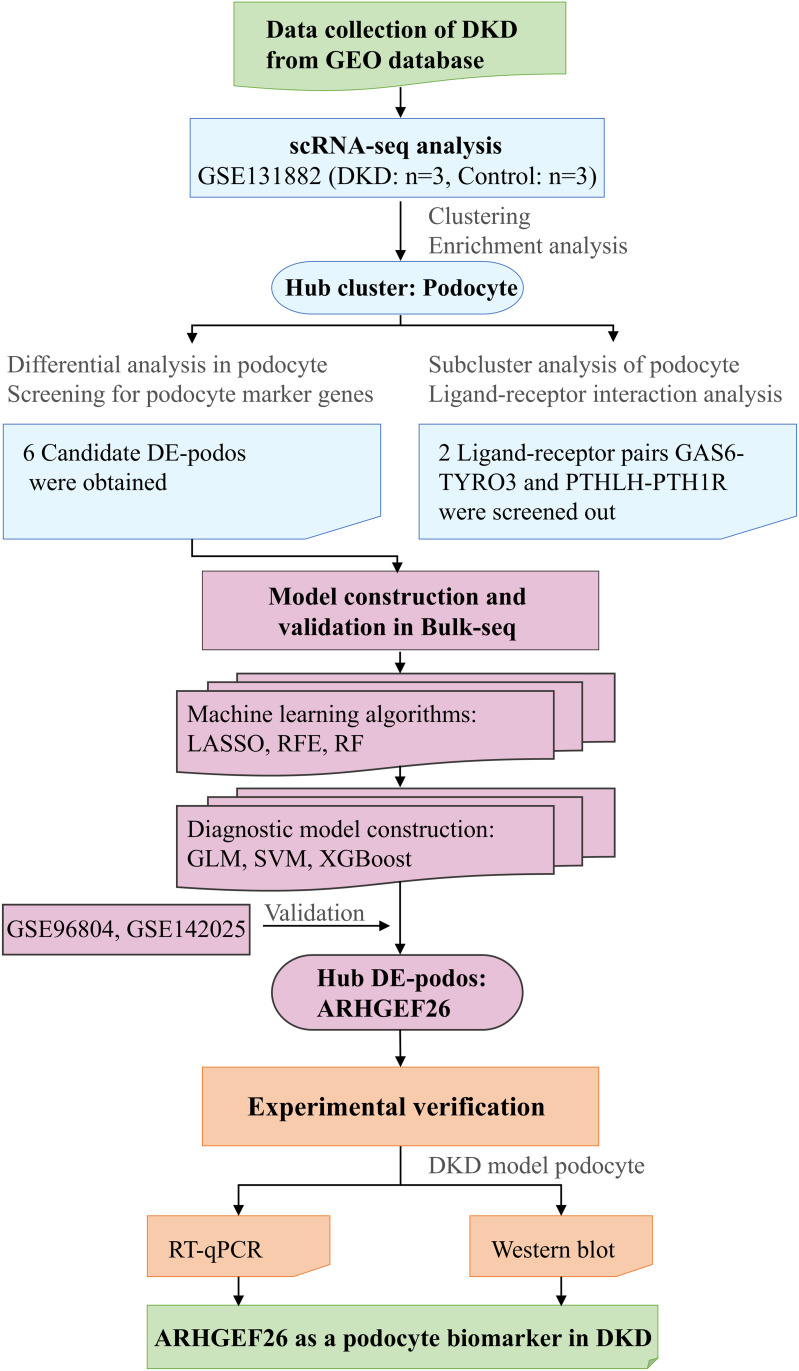
Workflow of the study. DE-podo, differentially expressed podocyte marker genes; LASSO, least absolute shrinkage and selection operator; RFE, recursive feature elimination; RF, random forest; GLM, generalized linear model; SVM, support vector machine; XGBoost, extreme gradient boosting; DKD: diabetic kidney disease; RT-qPCR, real-time quantitative polymerase chain reaction.

Herein, we identified differentially expressed podocyte marker genes (DE-podos) in DKD by analyzing the scRNA-seq (GSE131882) and bulk RNA-seq (GSE96804) datasets downloaded from the Gene Expression Omnibus (GEO) database. The biological functional characteristics of DKD and the cellular heterogeneity and functional differences of podocytes in DKD were elucidated through enrichment, clustering, and ligand–receptor (LR) interaction analyses. Multiple machine-learning algorithms were used to identify hub DE-podos and construct diagnostic models for DKD. The expression levels of hub DE-podos were validated using external datasets, reverse transcription quantitative real-time PCR (RT-qPCR) and Western blot to confirm the reliability of the results (Fig 1). These findings contribute to a better understanding of the role of podocytes in DKD, and may have implications for disease diagnosis and treatment.

## Results

### Identification of cell types

**Fig 2:**
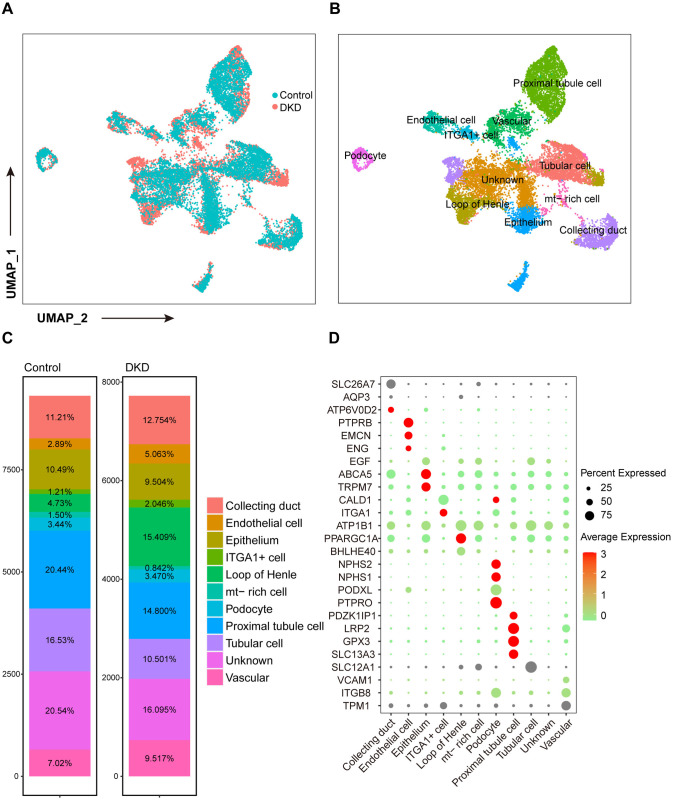
Dimension reduction and cell annotation in scRNA-seq GSE131882. (A) Cell distribution of the DKD and control groups in UMAP plot. (B) Ten cell types were annotated in scRNA-seq GSE131882, including collecting duct cell, endothelial cell, epithelial cell, ITGA1 + cell, loop of Henle, mt-rich cell, podocyte, proximal tubule cell, and vascular cell. (C) Proportions of each cell type in the DKD and control groups. (D) Expression levels of marker genes for annotation. scRNA-seq: single-cell RNA sequencing; DKD: diabetic kidney disease; UMAP: uniform manifold approximation and projection.

After filtering the single-cell dataset GSE131882, 17,035 cells were retained for analysis. Among them, 7723 cells belonged to the diabetic kidney disease (DKD) group and 9312 cells belonged to the control group (Fig 2A). Based on cell cluster-specific markers, 10 major cell types were identified: collecting duct cells, endothelial cells, epithelial cells, loop of Henle, podocytes, proximal tubule cells, and vascular cells. In addition, a subset of clusters with lower marker expression levels than those of all the other clusters was categorized as unknown. Notably, we identified a cluster with exclusive expression of mitochondrial genes, identified as mt-rich cells, and another cluster distinguished by high ITGA1 expression, categorized as ITGA1+ (Fig 2B). The respective proportions of each cell type of the DKD and control groups were represented via stack figure (Fig 2C). The proportions of proximal tubule cell and loop of Henle were significantly changed, while the proportion of podocyte did not show a significant difference. The marker genes used for annotation and their average expression levels in the different cell types are shown in Fig 2D. However, changes in cellular proportion and function remain to be further investigated to elucidate their potential role in the pathogenesis of DKD.

### Biological functional characteristics in diabetic kidney disease

**Fig 3:**
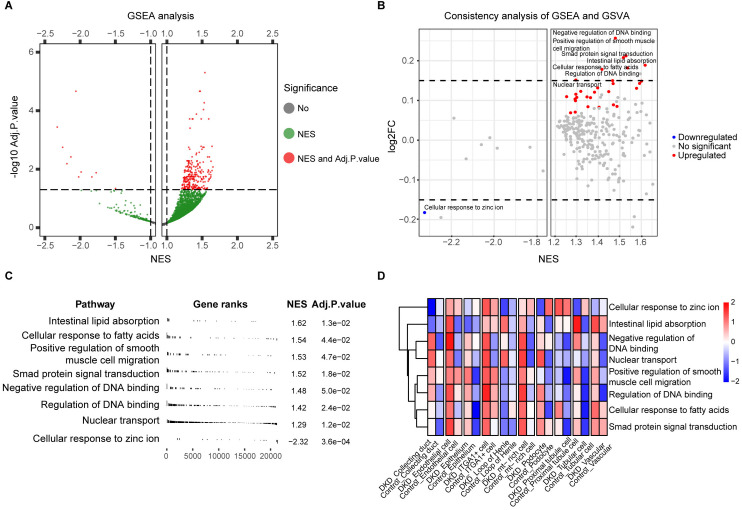
Characteristics of biological processes involved in DKD. (A) Volcano plot of differential BP in DKD identified by GSEA analysis. The grey dots represent the BP without significant differences, and green dots represent BP where only the NES value is significant, whereas red dots represent BP, in which both the NES value and Adj.P.value are significant. (B) Consistency analysis of GSEA and GSVA. Grey dots symbolize BP that did not meet the screening conditions (|log2FC| > 0.15), while red dots symbolize upregulated BP and blue dots symbolize downregulated BP. (C) List of NES value and Adj.P.value for the top 8 BP in GSEA. (D) GSVA scores of the top 8 BP presented in the heatmap. DKD: diabetic kidney disease; BP: biological processes; GSEA: gene set enrichment analysis; NES: normalized enrichment score; GSVA: gene set variation analysis.

Gene Set Enrichment Analysis revealed significant alterations in 268 biological processes (Adj. p < 0.05) (Fig 3A). After scoring and screening these 268 biological processes (BP) by Gene Set Variation Analysis (GSVA), 30 BP were identified, with 29 showing co-upregulation and one showing co-downregulation across both GSEA and GSVA (Fig 3B). Among them, eight BP showed significant differences between the DKD and control groups (|log2FC| > 0.15), including intestinal lipid absorption, cellular response to fatty acids, positive regulation of smooth muscle cell migration, Smad protein signal transduction, negative regulation of DNA binding, regulation of DNA binding, nuclear transport, and cellular response to zinc ions (Fig 3C, D). Notably, cellular response to zinc ions was the only co-downregulated gene set. The enrichment score was predominantly determined for genes encoding metallothioneins, including MT2A, MT1G, MT1E, MT1X, MT1H, MT1F, and MT1M. Metallothionein is a pivotal regulator of zinc ion homeostasis and is known to exert protective effects against cellular damage [21]. The expression levels of metallothionein are elevated in DKD, which may mitigate podocyte injury and ameliorate DKD progression [22,23]. Consequently, downregulation of the cellular response to zinc ions in DKD may be associated with alterations in the expression and functionality of metallothionein, concurrently affecting the cellular status of podocytes.

### Cellular heterogeneity and functional differences in diabetic kidney disease

**Fig 4:**
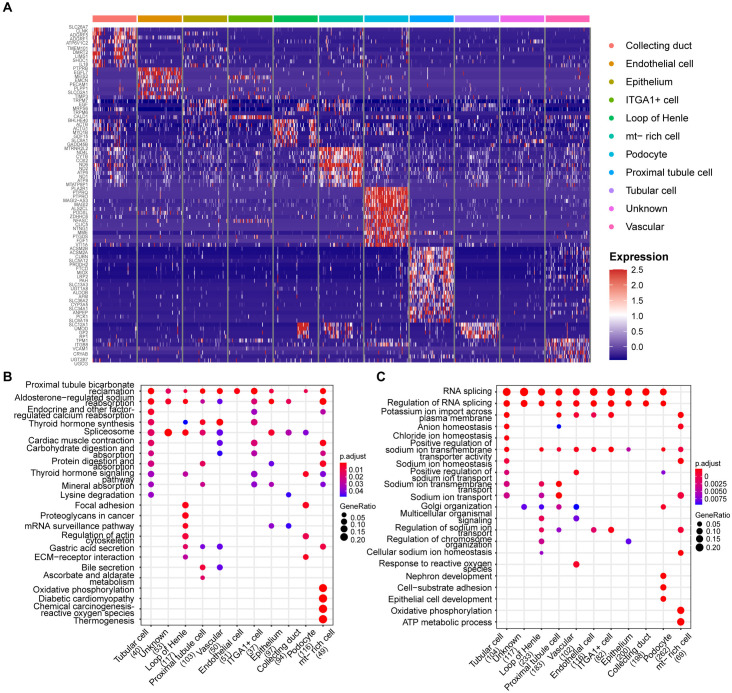
Cell-type marker genes and functional enrichment analysis. (A) Relative expression levels of representative cell-type marker genes were visualized in the heatmap. (B) KEGG pathway enrichment analysis of each cell type. (C) GO-BP enrichment analysis of each cell type. KEGG: kyoto encyclopedia of genes and genomes; GO-BP: gene ontology biological processes.

The expression levels of cell-type markers are shown in Fig 4A. Functional enrichment analysis indicated that marker genes of different cell types were enriched in several identical pathways or participated in the same biological processes, such as proximal tubule bicarbonate reclamation, aldosterone-regulated sodium reabsorption, RNA splicing, and positive regulation of sodium ion transmembrane transporter activity. However, certain cell types, particularly podocytes, execute unique biological functions that are not commonly performed by other cell types within the renal system. The results of Kyoto Encyclopedia of Genes and Genomes (KEGG) enrichment analysis revealed that focal adhesion, ECM–receptor interaction, and regulation of the actin cytoskeleton were specifically enriched in podocytes and loop of Henle (Fig 4B). Gene Ontology biological processes (GO-BP) enrichment analysis showed that nephron development, cell-substrate adhesion, and epithelial cell development were only enriched in podocytes (Fig 4C). These findings suggested that podocytes are vital for preserving the glomerular filtration barrier, and that their dysfunction is emerging as an early hallmark of DKD.

### Heterogeneity among podocytes

**Fig 5:**
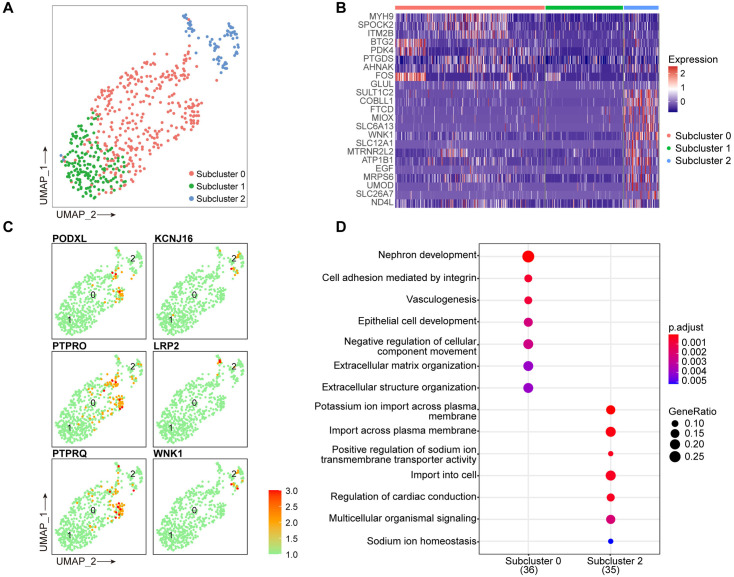
Unveiling cellular heterogeneity through podocyte subcluster analysis. (A) Podocytes were divided into three distinct subclusters, labeled as 0, 1, and 2. (B) The top ten cell cluster marker genes of each subcluster were selected for presentation of their expression level in the form of a heatmap. (C) Representative marker genes of subcluster 0 (PODXL, PTPRO, and PTPRQ) and subcluster 2 (KCNJ16, LRP2, and WNK1). (D) GO-BP enrichment analysis of subcluster marker genes. The top seven items were selected for presentation. GO-BP: gene ontology biological processes.

A total of 588 podocytes were identified, 268 of which belonged to the DKD group and 320 to the control group. Subsequent subcluster analysis delineated three distinct subclusters among the podocytes (Fig 5A, S1 Table). No upregulated marker genes were found in subcluster 1 (Fig 5B). Subcluster 0 highly expressed common podocyte markers such as PODXL and PTPRO, whereas subcluster 2 highly expressed marker genes such as KCNJ16, LRP2, and WNK1 (Fig 5C). In subcluster 0, the marker genes were enriched for nephron development, cell adhesion mediated by integrins, negative regulation of cellular component movement, and extracellular matrix organization. In subcluster 2, marker genes were enriched in the positive regulation of sodium ion transporter activity, potassium ion import across the plasma membrane, sodium ion homeostasis, and import into cells (Fig 5D). Taken together, it can be concluded that there was heterogeneity among the podocyte subclusters and that subcluster 0 was the main component of podocytes. Subcluster 0 podocytes were mainly responsible for the biological processes of podocyte adhesion, whereas subcluster 2 podocytes were mainly associated with ion transport and homeostasis. Concurrently, these findings implied that podocytes may be involved in the biological process of ion homeostasis imbalance in DKD.

### Pivotal ligand–receptor pairs in early diabetic kidney disease

**Fig 6:**
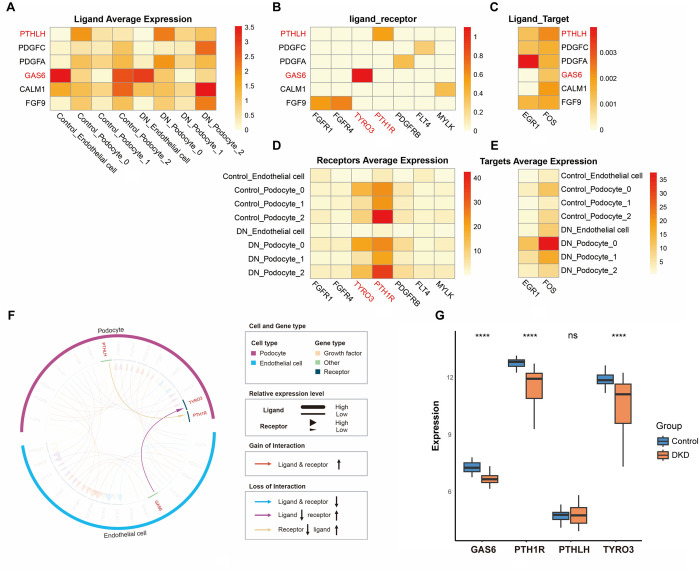
Possible LR interactions between endothelial cells and podocytes in DKD. (A) The average expression levels of ligands in endothelial cells and podocytes. (B) Confidence of LR interaction. The closer score to 1, the higher the credibility of interaction between that LR pair. (C) Ligands and their target genes. The heatmap denotes regulatory potential scores between ligands and targets. (D) The average expression levels of receptors in endothelial cells and podocytes. (E) The average expression levels of targets in endothelial cells and podocytes. (F) Two pairs of LRs and their corresponding ligand cell/receptor cell are presented in the form of an iTALK circos plot. Circos plots showing all putative gain or loss of cell–cell interaction events in DKD via LR pair signaling between podocyte subclusters and endothelial cells. (G) The expression levels of GAS6, PTH1R, PTHLH, and TYRO3 provided by a DKD-related glomerular bulk RNA-seq dataset GSE96804. *P < 0.05, **P < 0.01, ***P < 0.001, ****P < 0.0001 by Student’s t test. LR: ligand–receptor; bulk RNA-seq: bulk RNA sequencing.

Based on the fact that the intercellular crosstalk between podocytes and glomerular endothelial cells was essential for glomerular filtration maintenance [24,25], we analyzed the LR interactions between these cell types to identify novel LR pairs of podocytes involved in DKD progression. Because podocytes can be categorized into three subclusters, all podocyte subclusters and glomerular endothelial cells were used to investigate intercellular crosstalk. To identify high-confidence LR pairs, we employed the NicheNet algorithm, which integrates ligand activity, receptor expression, and literature support for ligand-receptor interactions to generate a composite score [26]. To further enhance the reliability of our candidate ligands, we selected ligands that have been experimentally validated to participate in glomerular cell interactions. Six ligands were screened: PTHLH, PDGFC, PDGFA, GAS6, CALM1, and FGF9 (Fig 6A). Their pairing receptors were PTH1R, FLT4, PDGFRB, TYRO3, MYLK, and FGFR1; furthermore, the target genes for these ligands were identified (Fig 6B, C). Among these, the average expression levels of TYRO3 and PTH1R were the highest (Fig 6D). Considering that high receptor expression levels may enhance intercellular crosstalk through LR interactions, GAS6–TYRO3 and PTHLH–PTH1R were chosen for further exploration.

The ligand growth arrest-specific gene 6 (GAS6) was mainly expressed in endothelial cells, whereas its pairing receptor, TYRO3 protein tyrosine kinase (TYRO3), was expressed in podocyte subclusters 0 and 2 (Fig 6A, D). The confidence level for the interaction between GAS6 and TYRO3 was extremely high, suggesting that endothelial cells probably communicate with podocytes via GAS6–TYRO3 (Fig 6B). Conversely, parathyroid hormone-like hormone (PTHLH) was mainly expressed in podocyte subcluster 0, whereas its pairing receptor, parathyroid hormone 1 receptor (PTH1R), was mainly expressed in podocyte subcluster 2, indicating that it may mediate intercellular crosstalk between podocyte subclusters (Fig 6A, D). Moreover, in the DKD group, the transcription factor FOS, a principal target gene of GAS6 and PTHLH, was significantly expressed in podocyte subcluster 0 (Fig 6E). FOS is involved in regulating cell proliferation, differentiation, and transformation. FOS expression is downregulated in DKD model cell lines [27]. Therefore, GAS6–TYRO3 and PTHLH–PTH1R may participate in DKD progression by regulating the target gene, FOS. In addition, the iTALK results suggested that the interaction between GAS6–TYRO3 and PTHLH–PTH1R was attenuated in DKD owing to the downregulation of GAS6 and PTH1R expression levels (Fig 6F). The DKD-related bulk RNA-seq validation dataset GSE96804 verified the downregulation of GAS6 and PTH1R, suggesting that GAS6–TYRO3 and PTHLH–PTH1R LR interactions may gradually weaken with DKD progression (Fig 6G).

### Identification of differentially expressed podocyte marker genes for diabetic kidney disease

**Fig 7:**
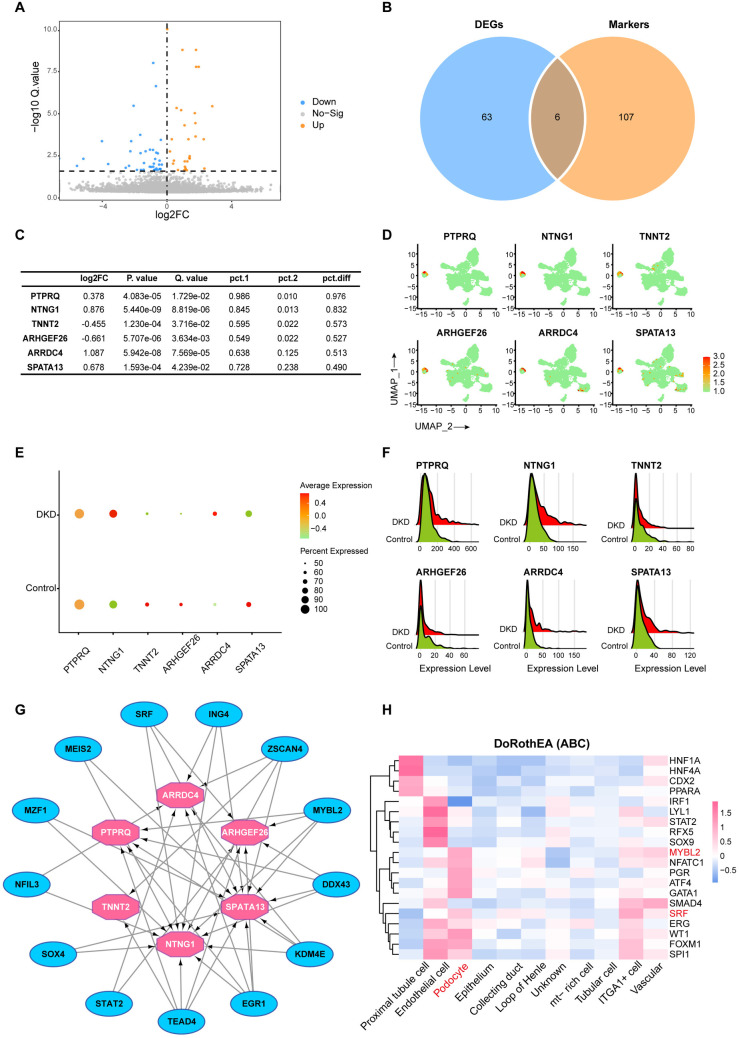
Differential expression analysis of podocytes in scRNA-seq. (A) Volcano plots of podocyte DEGs between the DKD group and control group. The grey dots represent the genes expressed without significant differences, and orange dots represent upregulated genes, whereas blue dots represent downregulated genes. (B) Identification of DE-podos with a Venn diagram. Blue represents DEGs, orange represents podocyte marker genes. (C) Information concerning DE-podos. pct.1, percentage of cells in which the gene is detected in podocytes; pct.2, percentage of cells in which the gene is detected in non-podocyte cells. pct.diff, difference between pct.1 and pct.2. (D) UMAP plot of DE-podos expression and cellular localization. Each dot corresponds to a single cell, with color intensity reflecting the relative gene-expression levels: red denotes high expression, while green signifies low expression. (E) Dot plot of the average expression levels of DE-podos. (F) Ridgeplot of the relative expression levels of DE-podos. (G) Identification of hub transcription factors targeting DE-podos using the Cytoscape plugin iRegulon. (H) Heatmap of the most variable TFs activity among different cell types. scRNA-seq: single-cell RNA sequencing; DEGs: differentially expressed genes; DE-podo: differentially expressed podocyte marker gene; UMAP: uniform manifold approximation and projection.

A total of 69 genes were screened as differentially expressed genes (DEGs) in podocytes under the conditions of Q.value < 0.05 (Fig 7A). In addition, 113 podocyte marker genes have been identified in a previous cluster analysis. Taken together, six genes (DE-podos) were screened when the DEGs intersected with podocyte marker genes (Fig 7B). To select a gene with the potential to serve as a podocyte marker, we investigated the degree of difference and specificity of these six genes. To quantitatively reflect specificity, we calculated the difference between podocytes and other kidney cell types that expressed DE-podos and noted the difference as pct.diff, while log2FC served as an indicator of the degree of difference between the DKD and control groups (Fig 7C). Moreover, the expression specificity, average expression level, and relative expression level of DE-podos were calculated and visualized (Fig 7D–F). The expression levels of PTPRQ, NTNG1, ARRDC4, and SPATA13 were upregulated, whereas those of TNNT2 and ARHGEF26 were downregulated in the DKD group. Among the DE-podos, PTPRQ showed the best specificity, with a high pct.diff of 97.6% (Fig 7C). However, the degree of difference in PTPRQ expression between the DKD and control groups was not particularly significant (log2FC = 0.378), indicating that PTPRQ is a stable marker for podocytes; however, its expression is less influenced by the state of DKD.

We then used the Cytoscape plugin iRegulon to identify the major transcription factors (TFs) targeting the above DE-podos, among which 13 TFs with normalized enrichment score (NES) ≥ 4 were used to construct the regulatory network (Fig 7G, S2 Table). To further validate the cell-specific regulatory activity of TFs, we evaluated TF activity in the scRNA-seq data using Dorothea algorithm. Results indicated that proximal tubule cells, endothelial cells and podocytes have high TF activity (Fig 7H). Notably, hub TFs MYBL2 and SRF were activated in podocyte and had a significant regulatory association with the identified DE-podos ARHGEF26, SPATA13 and NTNG1. These TFs are directly related to cell cycle regulation and actin cytoskeleton [28,29], and may cooperatively orchestrate podocyte proliferation, differentiation, and cytoskeletal remodeling in DKD through interactions with their target genes.

### Screening of hub differentially expressed podocyte marker genes with machine learning

**Fig 8:**
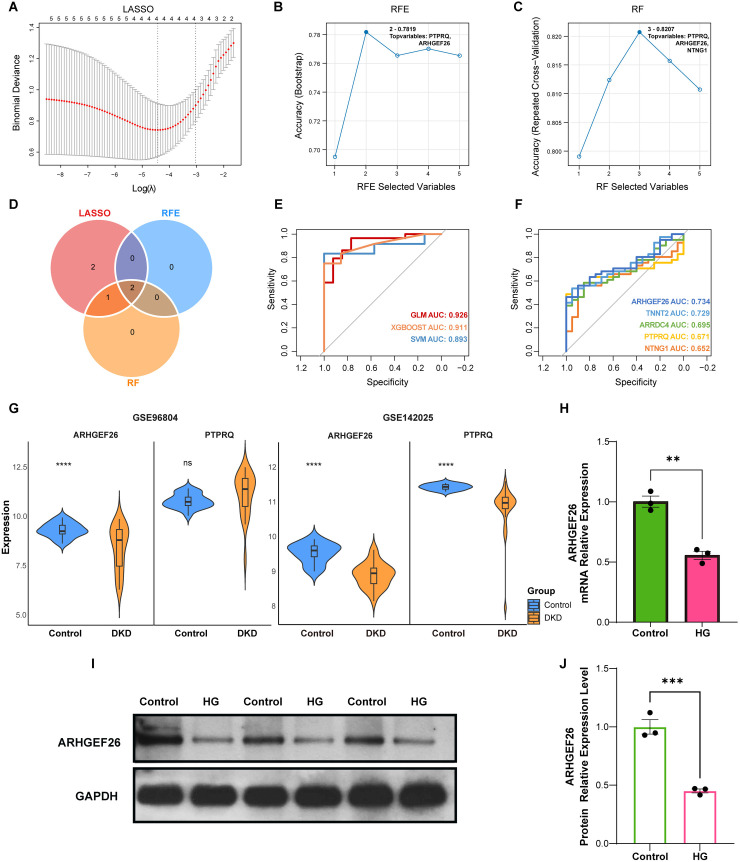
Identification and validation of hub DE-podos in bulk RNA-seq. (A) Selection of feature genes via LASSO regression. Four feature genes with non-zero coefficients were selected by an optimal lambda value. (B) Selection of feature genes via RFE algorithm. Two feature genes were selected by an optimal accuracy. (C) Selection of feature genes via RF algorithm. Three feature genes were selected by an optimal accuracy. (D) Venn diagram showing the intersections of hub DE-podos by LASSO, RFE, and RF. (E) ROC curves of diagnostic models constructed using two hub DE-podos based on GLM, XGBoost, and SVM algorithms. (F) ROC analysis of the diagnostic performance of six DE-podos. (G) Expression validation of hub DE-podos in GSE96804 dataset (left) and GSE142025 dataset (right). (H) RT-qPCR validation of ARHGEF26. (I-J) Western blot images and quantification of ARHGEF26 expression in podocytes treated with normal control and high-glucose (HG) medium. Data are represented as mean ± SEM. *P < 0.05, **P < 0.01, ***P < 0.001, ****P < 0.0001 by Student’s t test (G-J). DE-podo: differentially expressed podocyte marker gene; bulk RNA-seq: bulk RNA sequencing; LASSO: least absolute shrinkage and selection operator; RFE: recursive feature elimination; RF: random forest; ROC: receiver operating characteristic; GLM: generalized linear model; SVM: support vector machine; XGBoost: extreme gradient boosting; HG: high glucose.

Further analysis was conducted on the new bulk RNA-seq glomerular dataset GSE96804, which included 41 DKD and 20 control samples. Except for SPATA13, all other five DE-podos (Fig 7C) were verified in this external dataset. We employed a multi-algorithmic approach for feature gene screening using the least absolute shrinkage and selection operator (LASSO) regression, recursive feature elimination (RFE), and random forest (RF) algorithms in GSE96804. The LASSO regression identified four hub DE-podos with non-zero regression coefficients: PTPRQ, NTNG1, ARHGEF26, and ARRDC4 (Fig 8A). The RFE algorithm identified two hub DE-podos, PTPRQ and ARHGEF26, with a maximum accuracy of 0.7819 (Fig 8B). Finally, the RF algorithm identified three hub DE-podos, PTPRQ, ARHGEF26, and TNNT2, with a maximum accuracy of 0.8224 (Fig 8C). By intersecting the three algorithms, two hub DE-podos were identified: PTPRQ and ARHGEF26 (Fig 8D). Next, these two hub DE-podos were used to develop an optimal diagnostic model for DKD. Similarly, we used multiple machine-learning algorithms to construct the fitting models, including generalized linear model (GLM), support vector machine (SVM), and extreme gradient boosting (XGBoost). By calculating the receiver operating characteristic (ROC) curves and area under the curve (AUC) values, we observed that the GLM achieved the highest AUC value of 0.926. This was followed by the XGBoost model with an AUC of 0.911 and the SVM model with an AUC of 0.893 (Fig 8E). These results indicated the high efficacy of the diagnostic model developed based on the two hub genes.

Subsequently, we used ROC curves to investigate the association between DE-podo expression and DKD patient prognosis and evaluate their potential predictive usefulness in DKD. High diagnostic specificity and sensitivity for DKD were regarded as having an AUC greater than 0.700. According to Fig 8F, ARHGEF26 exhibited an AUC of 0.734, TNNT2 exhibited an AUC of 0.729, ARRDC4 exhibited an AUC of 0.695, PTPRQ exhibited an AUC of 0.671, and NTNG1 exhibited an AUC of 0.652. These findings demonstrated that ARHGEF26 has the best diagnostic value, suggesting that it may be a potent biomarker for DKD.

### Expression validation of hub differentially expressed podocyte marker genes for diabetic kidney disease

We validated the expression profiles of hub DE-podos in the DKD-related datasets GSE96804 and GSE142025. The results of GSE96804 showed no significant difference in the expression level of PTPRQ between the DKD and control groups (Fig 8G). Furthermore, because of the observed inconsistencies in PTPRQ expression across the GSE131882 (scRNA-seq) and GSE142025 (bulk RNA-seq of renal tissues) datasets, the role of PTPRQ in DKD remains inconclusive. Therefore, only ARHGEF26 exhibited consistent results across both the training and validation datasets, demonstrating a significant downregulation of ARHGEF26 in DKD renal glomerular tissues compared with that seen in control samples.

### Experimental validation of ARHGEF26 in podocytes treated with high-glucose

To confirm the expression of ARHGEF26 in DKD, we established an in vitro high glucose (HG) -induced mouse podocyte model. Our findings showed that the expression levels of ARHGEF26 were significantly reduced in the cells of HG group compared with those of the control group, which is consistent with previous results (Fig 8H). In addition, a significant decrease in ARHGEF26 protein level was observed in high-glucose treated podocytes, as shown by Western blot analysis (Fig 8I, J). This consistency suggested the potential role of ARHGEF26 as a novel biomarker for DKD.

## Discussion

Diabetic nephropathy, which constitutes the predominant cause of end-stage renal disease (ESRD), is a prevalent complication associated with diabetes, with an estimated 30%–50% of affected individuals ultimately advancing to ESRD [2,30]. Podocyte injury is a pivotal event in the pathogenesis of DKD, and podocytes, which are highly specialized glomerular epithelial cells, are essential for maintaining the integrity of the glomerular filtration barrier [31,32]. Understanding the mechanisms underlying podocyte injury is essential for developing more effective therapies for DKD and for potentially reducing the progression to ESRD. In the present study, an integrated bioinformatics analysis was conducted to determine the potential molecular mechanisms and biological processes of podocytes in DKD progression. The findings obtained herein revealed that the distinct biological processes altered in DKD are associated with podocytes, which exhibit cellular heterogeneity and functional differences. To explore podocyte-related biomarkers, we further identified DE-podos using comprehensive analysis and multiple machine-learning algorithms. Our findings indicated that ARHGEF26 is significantly downregulated in DKD, suggesting its potential as a podocyte-specific biomarker.

Proteinuria in patients with DKD is closely associated with podocyte dysfunction. Although our results did not reveal a significant difference in the proportion of podocytes between the DKD and control groups, this finding still holds important biological significance. Existing studies have shown that in the early stages of diabetic kidney disease, podocytes may initially undergo structural damage such as hypertrophy or compensatory changes, and then gradually undergo apoptosis and detachment as the disease progresses [33,34]. Therefore, we speculate that in the early stages of DKD, changes in podocyte proportion may not be significant, but their function may already be impaired. Through enrichment analysis, including GSVA and GSEA, we delineated the biological processes that were significantly altered in DKD. Notably, we observed that the cellular response to zinc ions was the only biological process downregulated in DKD. Among the genes contributing to the enrichment score, the metallothionein family is known for its role in modulating the intracellular concentrations of heavy metal ions, including zinc [21]. Recently, growing evidence has indicated that changes in ion channels are critical determinants of injury in DKD [35]. Among these, the zinc transporter 8 (ZnT8) protects against epithelial-to-mesenchymal tubulointerstitial fibrosis in DKD [36]. Metallothionein alleviates oxidative stress in the renal tissues of diabetic rats, thereby preventing the development of DKD [23]. Moreover, metallothionein overexpression reduces podocyte damage and ameliorates DKD [22]. Therefore, downregulation of the cellular response to zinc ions in DKD is probably associated with the altered expression and function of metallothionein, which in turn affects the cellular state of podocytes.

Diabetic nephropathy is characterized by dysfunction of the glomerular filtration barrier, which is composed of podocytes and glomerular endothelial cells (GECs) [32,37,38]. The impaired function of these two cell types is one of the major events in the early stages of DKD, and progressive podocyte loss is associated with podocyte–GEC crosstalk [39–42]. Based on these facts, we investigated the LR interactions between podocytes and GECs in DKD and identified two potential key LR pairs: GAS6–TYRO3 and PTHLH–PTH1R. The results of iTALK suggested a sustained loss of these LR interactions as DKD progressed. The binding of Gas6 and TYRO3 can activate downstream signaling pathways that regulate cellular processes, inflammation, and the immune response [43,44]. Tyro3 signaling in early DKD initially increases and then declines as DKD progresses [45]. TYRO3 is upregulated under high glucose stimulation and mediates anti-inflammatory responses, indicating its potential protective role in early DKD [46]. The interaction between PTHLH and PTH1R has been implicated in renal tubulointerstitial damage and glomerular diseases, including DKD [47]. A previous study showed that PTHLH and PTH1R are expressed in the glomeruli of streptozotocin-induced diabetic mice, and that the expression levels of PTHLH and PTH1R are upregulated in a mouse podocyte cell line induced by high glucose [48]. However, the specific roles and mechanisms of GAS6–TYRO3 and PTHLH–PTH1R interactions in DKD remain unclear.

To further identify podocyte-related biomarkers in DKD, we conducted a comprehensive analysis and screening and found that ARHGEF26 was downregulated in both the glomeruli and podocytes, emphasizing its potential role in DKD progression. ARHGEF26, also known as SGEF, encodes Rho guanine nucleotide exchange factor 26, which mediates the activation of Rho proteins [49]. ARHGEF26 specifically activates RhoG protein and promotes macropinocytosis, a key process in cellular endocytosis and signal transduction [50]. In glomerular vascular endothelial cells, ARHGEF26 modulates angiogenesis by promoting macropinocytosis of vascular endothelial growth factor receptor 2 (VEGFR2) [51]. VEGF is prevalent in various organs and tissues, including the heart, kidneys, and lungs. Research in a type 1 diabetic mouse model has indicated that a local decrease in glomerular vascular endothelial growth factor A (VEGFA) exacerbates endothelial damage and hastens glomerular injury progression [52]. This implies that elevated VEGFA levels in diabetic kidneys may protect the microvasculature from harm [53,54]. Therefore, the expression of ARHGEF26 in glomerular endothelial cells may facilitate VEGFA-induced VEGFR-2 internalization on the cell surface via macropinocytosis, thereby protecting the glomerular microvasculature in diabetes. Downregulation of ARHGEF26 could affect angiogenic signaling pathways and potentially indicate a diminished capacity for renal repair and regeneration. Conversely, in epithelial cells, ARHGEF26 is expressed and contributes to the regulation of cell–cell interactions, specifically at tight junctions (TJs) and adherens junctions (AJs), which are crucial for maintaining the barrier function [55]. ARHGEF26 plays a key role in coordinating junction assembly and actomyosin contractility by binding Scribble and Dlg1, and activating RhoG to target intercellular junctions [56]. Therefore, the downregulation of ARHGEF26 in podocytes, which serve as specialized epithelial cells of the glomerulus, may lead to the disruption of TJs and AJs, potentially affecting the barrier function of podocytes and the filtration function of the glomerulus. Furthermore, the regulatory role of ARHGEF26 in cellular morphology and actomyosin contractility is crucial for podocyte health and function, thereby influencing the glomerular structure and filtration capacity. In conclusion, downregulation of ARHGEF26 in podocytes and glomeruli could potentially impair renal microvasculature and alter podocyte morphology and barrier function, consequently compromising the integrity of the glomerular filtration barrier and hastening DKD progression.

## Conclusion

**Fig 9:**
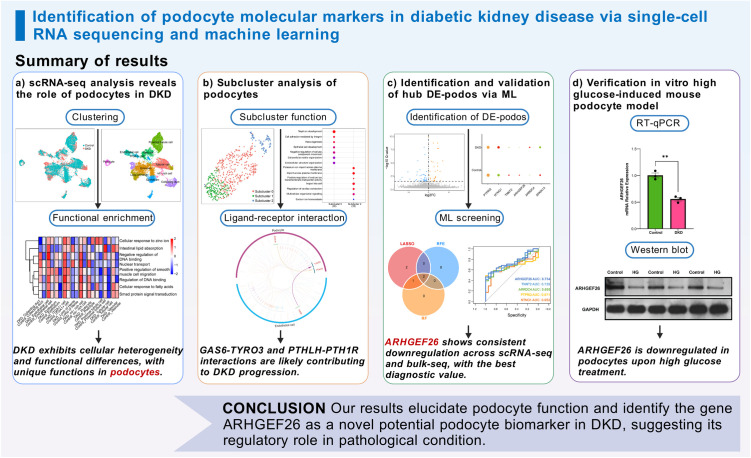
Graphical summary of results in this study.

This study focused on identifying potential podocyte biomarkers in DKD via single-cell RNA sequencing and machine learning, offering novel insights into diagnostic and therapeutic approaches to DKD. Our results elucidated podocyte function and identified the gene ARHGEF26 as a novel potential podocyte biomarker in DKD, suggesting its regulatory role in pathological conditions (Fig 9). Future research should refine the experimental design and explore the specific molecular mechanisms of ARHGEF26 in DKD.

## Materials and methods

### Data collection and quality control

The dataset of single-cell RNA sequencing of early human diabetic nephropathy, GSE131882, containing single-nucleus transcriptomic data of three normal controls and three patients with early DKD, was downloaded from the Gene Expression Omnibus (GEO) database (http://www.ncbi.nlm.nih.gov/geo) (S3 Table). Quality control analysis was performed on the data using the Seurat R package, with a filter set at min.cells = 3 and min.features = 500. Separated seurat objects were merged together using Merge function. Additionally, filtering based on the scRNAstat R package was applied to filter low-quality data. Gene Ensembl IDs were mapped to their corresponding official gene names using the org.Hs.e.g.,db R package.

### Dimensionality reduction and clustering

Principal component analysis with the parameter “npcs” set to 40 was performed using the RunPCA function. The nearest-neighbor search was performed using the FindNeighbours function, setting the parameter dims to 1:20. Distinct clusters were identified using the FindClusters function at a resolution parameter of 0.6. T-distributed stochastic neighbor embedding (tSNE) and uniform manifold approximation and projection (UMAP) were conducted for dimensionality reduction via the RUNTSNE and RunUMAP functions, with parameter dims set to 1:20. Cluster marker genes were identified by the FindAllMarkers function with min.pct = 0.5 and test.use = “roc”. Cell types were manually annotated based on previous studies and the CellMarker database. Additionally, cell-type marker genes were screened by the FindAllMarkers function with min.pct = 0.5 and test.use = “wilcox”.

### Gene set enrichment analysis and gene set variable analysis

To preliminary discover differences of biological processes (BP) between the DKD group and control group, the R packages fgsea and msigdbr were used for Gene Set Enrichment Analysis (GSEA). The reference gene sets were “c5.BP.v7.1. symbols” obtained from the Molecular Signature Database (MsigDB, https://www.gsea-msigdb.org/) for subsequent Gene Ontology biological processes (GO-BP) enrichment analysis. Gene sets with Adj.P.value < 0.05 were statistically significant and were used for further analysis. The results were visualized using the Enhanced Volcano R package.

To gain a deeper understanding of the variations among distinct cell types, we employed Gene Set Variable Analysis [57] (GSVA) using the GSVA R package. The average gene expression matrix was extracted as input data for GSVA using the average expression function. Thereafter, GSVA was conducted by the GSVA function, setting parameter method to “gsva” and “min.sz” to 10. The limma R package was used to analyze the differences in each gene set between the DKD and control groups. The output data were a score matrix the columns of which were cell types and rows were gene sets. Gene sets were considered statistically significant based on consistent upregulation or downregulation across both GSEA and GSVA, as indicated by the Normalized Enrichment Scores (NES) in GSEA and log2FC in GSVA. The results were visualized using plotGseaTable and heatmap functions.

### Functional enrichment analysis

The clusterProfiler R package was used for the functional enrichment of cell-type marker genes. Kyoto Encyclopedia of Genes and Genomes (KEGG) pathway enrichment analysis was performed using the compareCluster function, with a p-value cutoff of 0.05. GO-BP enrichment analysis was also conducted by the compareCluster function, with pAdjustMethod = “BH” and qvalueCutoff = 0.05. Redundant results of the GO-BP enrichment analysis were eliminated using the simplify function. Pathways were considered to indicate significant gene enrichment at p < 0.05. Functional enrichment results were visualized using the dot plot function.

### Clustering and functional analysis of cellular subclusters

Podocytes were selected for subcluster and marker gene function analyses. Distinct subclusters were identified using the FindClusters function with parameter resolution set to 0.2 and dims set to 1:10. Finally, GO and KEGG enrichment analyses were performed for each subcluster using the clusterProfiler R package.

### Ligand–receptor interaction analysis

The nichenetr R package was used to predict ligand–receptor (LR) interactions, target genes regulated by LR genes, and their expression in specific cell types. Podocytes and glomerular endothelial cells were designated as signaling and receiving cells, respectively, for their roles in glomerular filtration and microenvironmental communication. All parameters were set to default values. Ligand–receptor pairs validated by laboratory experiments or biochemical data were selected to construct matrices, one for LR interactions and another for regulatory scores between active ligands and their predicted targets. In addition, the average expression matrices of ligands, receptors, and targets were calculated. LR interaction analysis was performed using the iTALK R package. The FindLR function was used to analyze the differentially expressed genes between all subclusters of podocytes and endothelial cells and to predict the LR pairs between them. Subsequently, the LR pairs that were both in the results of nichenet and iTALK were selected and visualized using the LRPlot function.

### Differential expression and transcription factor analysis

Podocytes were extracted from the seurat object, and differential expression analysis was performed using the DESinger R package. A Q.value < 0.05 for Differentially expressed genes (DEGs) was considered statistically significant. By intersecting DEGs with known markers, differentially expressed podocyte marker genes (DE-podos) were identified that exhibited significant changes in expression between the DKD and control groups, representing the specific characteristics of podocytes.

Subsequently, Cytoscape plugin iRegulon was used to identify master regulators that targeted these DE-podos [58]. In this study, transcription factors (TFs) with normalized enrichment score (NES) ≥ 4 were retained to build the regulatory network, and other default parameters were left unchanged.

DoRothEA was used to evaluate TF activity of various cell types in scRNA-seq data [59]. Transcription factor regulatory networks that exhibited higher confidence levels (from A to C) supported by evidence were extracted and 20 TFs with the greatest variations were displayed. The scale method was used to measure the viper scores.

### Identification and validation of hub DE-podos based on machine learning

We analyzed the RNA expression profiles of the selected DE-podos in the GSE96804 dataset, which included 41 DKD and 20 control samples. The samples from the datasets were glomeruli from patients with DKD and normal portions of tumor nephrectomies (control group) (S3 Table). To select the optimal variables, the least absolute shrinkage and selection operator (LASSO) logistic regression model with 10-fold cross-validation was implemented using the glmnet R package, a recursive feature elimination (RFE) algorithm with bootstrap validation was performed using the Caret package, and a random forest (RF) algorithm with 10-fold cross-validation was performed using the randomForest R package. To validate the diagnostic value of the selected genes, the receiver operating characteristic (ROC) curve of each gene was calculated using the pROC package, which reflects the prediction accuracy of each selected factor in sample classification.

Hub genes identified by the three algorithms were selected to develop an optimal diagnostic model for DKD. The fitted model was subsequently constructed by combining a pair of non-integrated machine learning methods, the generalized linear model (GLM) and support vector machine (SVM), with an integrated algorithm, extreme gradient boosting (XGBoost). The receiver operating characteristic (ROC) curve and average optimism of the area under the curve (AUC) were calculated using the caret package to quantify the predicted probabilities of the model. Furthermore, the expression levels of the identified hub DE-podos were validated in GSE96804 and GSE142025 (which included 28 DKD kidney tissue samples and nine normal tissue samples).

### Real-time quantitative polymerase chain reaction (RT-qPCR)

The MPC-5 mouse renal podocyte cell line was utilized as the experimental model in this research, generously provided by Professor Jochen Reiser from the Rush University Medical Center (Chicago, IL, USA). The cells were cultured in glucose-free Dulbecco’s Modified Eagle Medium (DMEM, #11966025, Gibco, USA) supplemented with 10% fetal bovine serum (FBS; BI) and 1% penicillin/streptomycin at 37 °C under an atmosphere containing 5% CO_2_. Control cells were cultured in normal medium containing 5.5 mM glucose, whereas the high-glucose (HG) group cells were treated with 30 mM glucose for 24 h. Cells were subjected to overnight starvation from glucose before the high-glucose treatment.

RNA was extracted using an Ultrapure RNA Kit (CW0581M, CWBIO), followed by reverse-transcription to obtain cDNA. Quantitative real-time PCR was performed using the HiScript II Q RT SuperMix for qPCR (+gDNA wiper) kit (R223-01, Vazyme) and a CFX Connect™ Real-Time PCR Detection System (Bio-Rad, Shanghai). All procedures strictly adhered to the manufacturer’s instructions. The data were normalized and analyzed using the 2^−ΔΔCt^ method, with gene expression being normalized to β-actin. The primer sequences are provided below: ARHGEF26: forward, TCTTACAGAAGGGCAGTGGTCA and reverse, TTCAGGACAGGCTGGGACAT; β-actin: forward, AGGGAAATCGTGCGTGAC and reverse, CATACCCAAGAAGGAAGGCT.

### Western blot analysis

Proteins were extracted using RIPA lysis buffer (Solarbio, R0020), separated by SDS-PAGE, and transferred to PVDF membranes. The antibodies used were as follows: anti-ARHGEF26 (12493–1-AP, ProteinTech, USA) and anti-GAPDH (ab181602, Abcam, UK). Chemiluminescence was detected and images were captured. The relative expression level of ARHGEF26 was normalized to that of GAPDH for analysis.

### Statistical analysis

The mean ± SEM was utilized for expressing the data. Statistical analyses were conducted using GraphPad Prism 9.5.0 and R (version 4.3.1). Graphical summary was created with BioGDP (https://gdp.rjmart.cn). Student’s t-test was used to compare data between two groups, with the significance level set at P < 0.05. The levels of significance were denoted as follows: * P < 0.05, ** P < 0.01, *** P < 0.001, and **** P < 0.0001.

## Supporting information

S1 Table The number and percentage of podocyte subclusters in DKD group and control group. (DOCX)

S2 Table Regulatory network of target genes and transcription factors. (DOCX)

S3 Table The overview of the datasets. (DOCX)

S1 Raw_images All original immunoblot images. (PDF)
