# Supplementary material for: Identification of podocyte molecular markers in diabetic kidney disease via single-cell RNA sequencing and machine learning
Source: PLoS One. 2025 Jul 21;20(7):e0328352. doi: 10.1371/journal.pone.0328352 (PMC12279108; doi:10.1371/journal.pone.0328352)
Supplement: S1 Table — (DOCX) [file pone.0328352.s001.docx]

**S1 Table. The number and percentage of podocyte subclusters in DKD group and control group.**

|  | Subcluster 0 | Subcluster 1 | Subcluster 2 | Total |
| --- | --- | --- | --- | --- |
| DKD | 162 (60.45%) | 80 (29.85%) | 26 (9.70%) | 268 |
| Control | 174 (54.375%) | 94 (29.375%) | 52 (16.25%) | 320 |
