# Supplementary material for: Identification of podocyte molecular markers in diabetic kidney disease via single-cell RNA sequencing and machine learning
Source: PLoS One. 2025 Jul 21;20(7):e0328352. doi: 10.1371/journal.pone.0328352 (PMC12279108; doi:10.1371/journal.pone.0328352)
Supplement: S2 Table — (DOCX) [file pone.0328352.s002.docx]

**S2 Table. Regulatory network of target genes and transcription factors.**

| Transcription Factor | NES | Target Gene |
| --- | --- | --- |
| EGR1 | 6.911 | ARRDC4, ARHGEF26, SPATA13, NTNG1, PTPRQ |
| KDM4E | 5.667 | ARRDC4, SPATA13, NTNG1, PTPRQ |
| TEAD4 | 5.044 | ARHGEF26, SPATA13, NTNG1, TNNT2, PTPRQ |
| SRF | 4.761 | ARHGEF26, SPATA13, NTNG1 |
| STAT2 | 4.734 | ARRDC4, ARHGEF26 |
| ING4 | 4.600 | ARRDC4, SPATA13, NTNG1 |
| MYBL2 | 4.531 | ARHGEF26, SPATA13, NTNG1, PTPRQ |
| MEIS2 | 4.387 | SPATA13, NTNG1 |
| NFIL3 | 4.374 | ARRDC4, NTNG1 |
| DDX43 | 4.322 | ARHGEF26, SPATA13, NTNG1, PTPRQ |
| ZSCAN4 | 4.235 | ARRDC4, SPATA13, NTNG1, TNNT2 |
| MZF1 | 4.104 | SPATA13, NTNG1 |
| SOX4 | 4.021 | SPATA13, NTNG1 |
