# Supplementary material for: Identification of podocyte molecular markers in diabetic kidney disease via single-cell RNA sequencing and machine learning
Source: PLoS One. 2025 Jul 21;20(7):e0328352. doi: 10.1371/journal.pone.0328352 (PMC12279108; doi:10.1371/journal.pone.0328352)
Supplement: S3 Table — (DOCX) [file pone.0328352.s003.docx]

**S3 Table. The overview of the datasets.**

| Accession | Database | RNA Library | Sample size | Source |
| --- | --- | --- | --- | --- |
| GSE131882 | GEO | Single-cell RNA sequencing | Control: 3, DKD: 3 | Kidney tissue |
| GSE96804 | GEO | Bulk RNA sequencing | Control: 20, DKD: 41 | Kidney glomeruli |
| GSE142025 | GEO | Bulk RNA sequencing | Control: 9, DKD: 28 | Kidney tissue |
