# Supplementary figures and images for: Identification of podocyte molecular markers in diabetic kidney disease via single-cell RNA sequencing and machine learning
Source: PLoS One. 2025 Jul 21;20(7):e0328352. doi: 10.1371/journal.pone.0328352 (PMC12279108; doi:10.1371/journal.pone.0328352)

**Fig 8I**

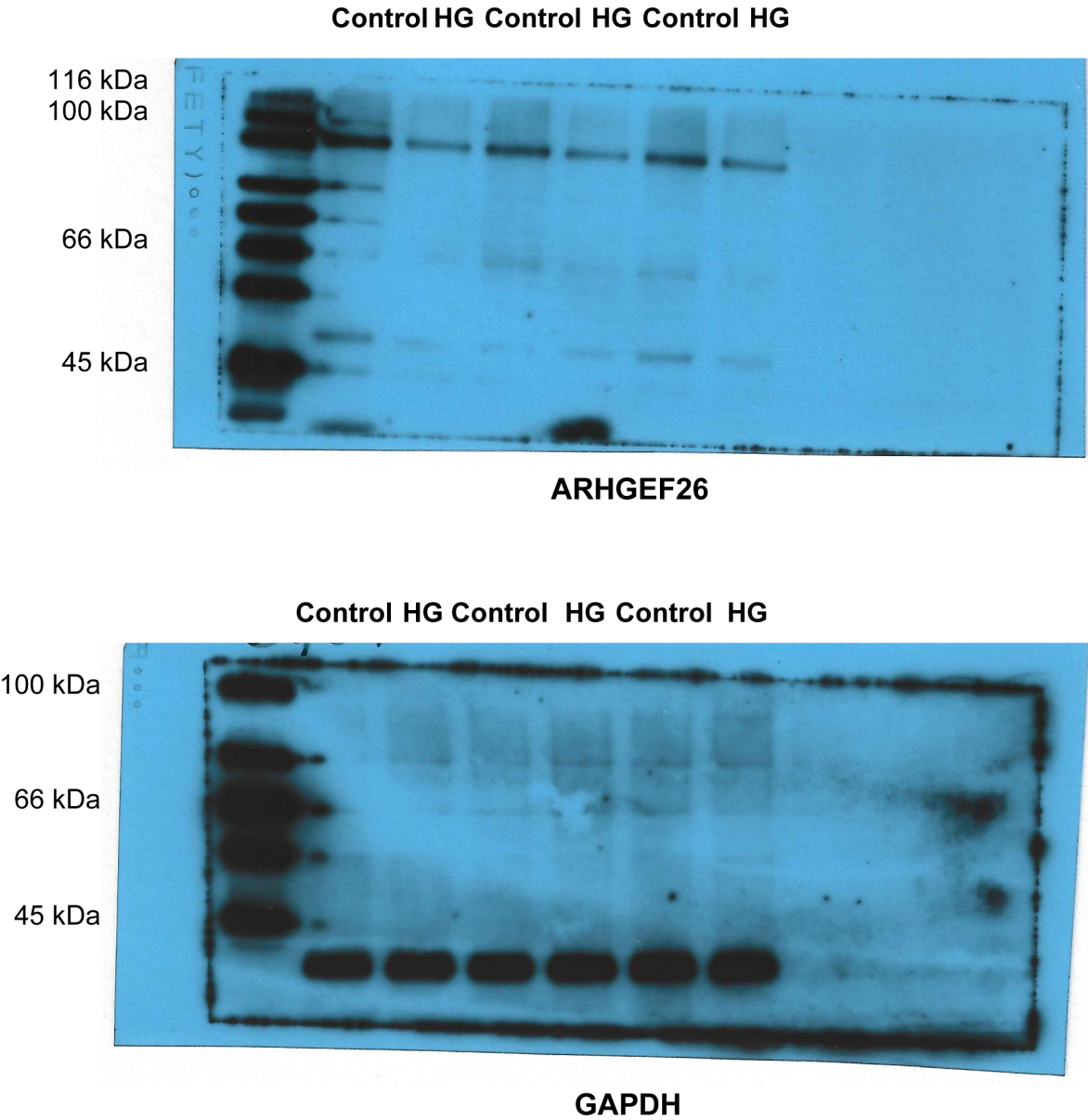

Supplement: S1 Raw_images — (PDF) [file pone.0328352.s004.pdf]
